# Diet-derived antioxidants and osteoporosis: A Mendelian randomization study

**DOI:** 10.1371/journal.pone.0293145

**Published:** 2023-11-29

**Authors:** Haitao Li, Lanlan Chen, Chaofeng Yuan, Hongqun Yang, Zhuangzhuang Ma, Jianlin Zuo

**Affiliations:** 1 Department of Orthopeadics, The China-Japan Union Hospital of Jilin University, Changchun, Jilin, China; 2 Department of Hepatobiliary and Pancreatic Surgery, General Surgery Center, The First Affiliated Hospital of Jilin University, Changchun, Jilin, China; 3 Department of Gastrointestinal Colorectal Surgery, The China-Japan Union Hospital of Jilin University, Changchun, Jilin, China; 4 Department of Orthopeadics, China-Japan Union Hospital of Jilin University, Changchun, Jilin, China; Nanjing Medical University, CHINA

## Abstract

**Background:**

Antioxidants can prevent osteoporosis, but the association between serum antioxidants and the cause of osteoporosis remains unknown. We aimed to utilize Mendelian randomization (MR) to determine whether genetically predicted serum levels of diet-derived antioxidants can affect the risk of osteoporosis, to determine the effect of dietary supplementation of antioxidants.

**Methods:**

Genetic variants associated with diet-derived antioxidants were selected from the genome-wide association studies. A total of 12,946 osteoporosis cases and 506,624 healthy controls were obtained from UK Biobank (UKB) and Genetic Factors of Osteoporosis (GEFOS) consortia. We implemented a two-sample MR design and performed several sensitivity analyses to evaluate the causal relationship.

**Results:**

In UKB, the genetically predicted higher β-carotene (OR = 0.863, p = 7.37 × 10^−6^, power = 100%) and γ-tocopherol (OR = 0.701, p = 0.021, power = 5%) had an inverse relationship with osteoporosis. However, only the association of serum β-carotene passed FDR correction. In GEFOS, there were no significant diet-derived antioxidants. The direction of the association of β-carotene with osteoporosis (OR = 0.844, p = 0.106, power = 87%) was consistent with that in the UKB dataset. A fixed-effects meta-analysis confirmed that β-carotene (OR = 0.862, p = 2.21 × 10^−6^) and γ-tocopherol (OR = 0.701, p = 2.31 × 10^−2^) could decrease the risk of osteoporosis. To reduce exclusion limit bias, we used total body bone mineral density, lumbar spine bone mineral density and femoral neck bone mineral density as surrogates and found that the genetically elevated circulating β-carotene level could increase total body BMD (beta = 0.043, p-value = 8.26 x 10^−5^, power = 100%), lumbar spine BMD (beta = 0.226, p-value = 0.001, power = 100%) and femoral neck BMD(beta = 0.118, p-value = 0.016, power = 100%).

**Conclusions:**

We observed that genetically predicted serum β-carotene could elevate BMD and prevent osteoporosis.

## Introduction

Osteoporosis is a highly prevalent disease estimated to affect 200 million people worldwide, especially those aged over 60 years [1]. Furthermore, the prevalence of osteoporosis and its complications is increasing with the rapid aging of the population globally, placing considerable physiological and financial burden on patients and society [2]. Thus, it is of importance to find effective ways to prevent the pathogenesis of osteoporosis.

Osteoporosis occurs due to the imbalance between activities of osteoclasts and osteoblasts, which is thought to contribute to the progressive reduction in bone quantity, mass, and strength, leading to an increased risk of fracture even caused by very-low-energy trauma [3]. Several risk factors have been identified for osteoporosis, including old age, pathological hormonal changes, calcium imbalance, medication history, inflammatory arthropathy, hematopoietic disorders, and so on. Additionally, recent studies have implicated oxidative stress in the pathogenesis of osteoporosis via the uncoupling of osteoclasts and osteoblasts function [4–7]. Such uncoupling can be achieved through the promotion of osteoclastogenesis, inducing apoptosis of osteoblasts and osteocytes, downregulating osteoprogenitor differentiation to the osteoblast cell lineage, and inactivating osteoblasts to influence bone mineral density (BMD) by pathways of bone metabolism [3]. Consequently, much research has been conducted to determine whether dietary supplementation of antioxidant-rich foods can ameliorate oxidative stress to improve bone density as preventive and therapeutic interventions in clinical applications. In a epidemiologic study of 56,736 women, Warensjö Lemming et al. showed that a diet consisting of more vegetables, fruits, and cereals can reduce the risk of fracture [8]. Additional human studies have also shown similar conclusions: whole plant foods rich in natural antioxidants can improve BMD [9, 10].

However, most of these studies are cross-sectional designs, whose data are from food frequency and diet recall questionnaires. Such studies can provide clinical associations while failing to give causation. Although large-scale randomized controlled trials (RCTs) are the gold standard for establishing causal relationships, they are expensive to run and many researchers may not have the opportunity to conduct them [3].

Mendelian randomization (MR) is an emerging approach in epidemiology used for causal inference, which uses genetic variants as the instrumental variables to estimate the causal effect of exposure on outcome. Apart from that, MR can reduce the bias caused by confounders or reverse causation since genetic variants are randomly allocated at conception [11].

We aimed to utilize MR to determine whether genetically predicted serum levels of diet-derived antioxidants can affect the risk of osteoporosis, to determine the effect of dietary supplementation of antioxidants. The causal relationship between five main dietary-derived antioxidants and osteoporosis was estimated using MR, hoping to give proper guidance on the prevention of osteoporosis.

## Materials and methods

### Data source description

Initially, we screened for all publicly available genome-wide association study (GWAS) summary statistics for diet-derived antioxidants, including carotene (lycopene, β-carotene), retinol, vitamin C (ascorbate), and vitamin E (α-tocopherol, γ-tocopherol). Frozen blood samples are assayed for serum antioxidants, by reverse-phase high-pressure liquid chromatography, gas chromatography separation and tandem mass spectrometry. The genetic associations with serum lycopene were obtained from a GWAS with adjustment of age, sex, and body mass index (BMI) in 441 adults [12]. The average age is 43.1±13.0 years old. The selected five SNPs were significant (p-value < 1 x 10^−6^) and suggested possible biological meanings, such as SNP rs7680948 located in the SETD7 gene. [12]. The genetic instruments for serum β-carotene were extracted from a GWAS meta-analysis in ~3,000 individuals, adjusting for age and sex (p-value < 1 x 10^−5^, LD R2 < 0.2) [13]. The average age is 57.8±6.8 years old. The genetic associations with serum retinol were derived from one source, which was the absolute value of circulating retinols from a GWAS in 5,006 Caucasian individuals (p-value < 5 x 10^−8^, LD R2 < 0.01), adjusting for cancer case status, age at blood collection, BMI, serum cholesterol, and study and genetic components [14]. The average age is 60.3±5.6 years old.

The genetic associations with vitamin C were extracted from a GWAS database. This GWAS consisting of 52,018 European individuals adjusting for age, sex, and study center, which was used as the main analysis, and the SNPs were selected based on p-value < 5 x 10^−8^ and LD R2 < 0.01 [15]. The average age is 59.5±19.5 years old.

For vitamin E, α-tocopherol and γ-tocopherol were assessed. Two GWASs of α-tocopherol were included where one represented the absolute level of circulating α-tocopherol and the other was the normalized value. The former GWAS included 5,006 Europeans and the SNPs were selected using p-value < 5 x 10^−8^ and LD R2 < 0.01 [16]. The average age is 53±32 years old. The latter GWAS included 7,725 European individuals, representing the α-tocopherol metabolite [17]. The γ-tocopherol GWAS was from the same study of the α-tocopherol metabolite. The instrumental variables (IVs) of α-tocopherol and γ-tocopherol metabolites were selected based on p-value < 1 x 10^−5^ and LD R2 < 0.01. The average age is 61±16 years old.

The genetic associations with osteoporosis were acquired from UKB and Genetic Factors of Osteoporosis (GEFOS) consortia. The UKB GWAS of osteoporosis was made up of 7,547 cases and 455,386 controls, adjusting for first 20 principal components, sex, age, age squared, interaction between sex and age, and interaction between sex and age squared. The GEFOS GWAS consisted of 5,399 cases and 51,238 controls, adjusting for genetic relatedness, sex, and age. We included the total body BMD GWAS summary statistics, lumbar spine BMD GWAS summary statistics and femoral neck BMD GWAS summary statistics as surrogates for osteoporosis. The BMD in our study is the result of DXA test and is standardized. According to the WHO Expert Committee, Criteria for osteoporosis is BMD < −2.5 SD t-score. The GEFOS GWAS of the total body BMD recruited 56,284 individuals from European adjusting for genetic relatedness, sex, and age. The GEFOS GWAS of the lumbar spine BMD consisted of 28,498 European individuals, adjusting for genetic relatedness, sex, and age. The GEFOS GWAS of the femoral neck BMD recruited 32,735 European individuals, adjusting for genetic relatedness, sex, and age. This MR study was performed using de-identified GWAS summary statistics, and ethical approval was obtained by each GWAS.

### MR design

**Fig 1:**
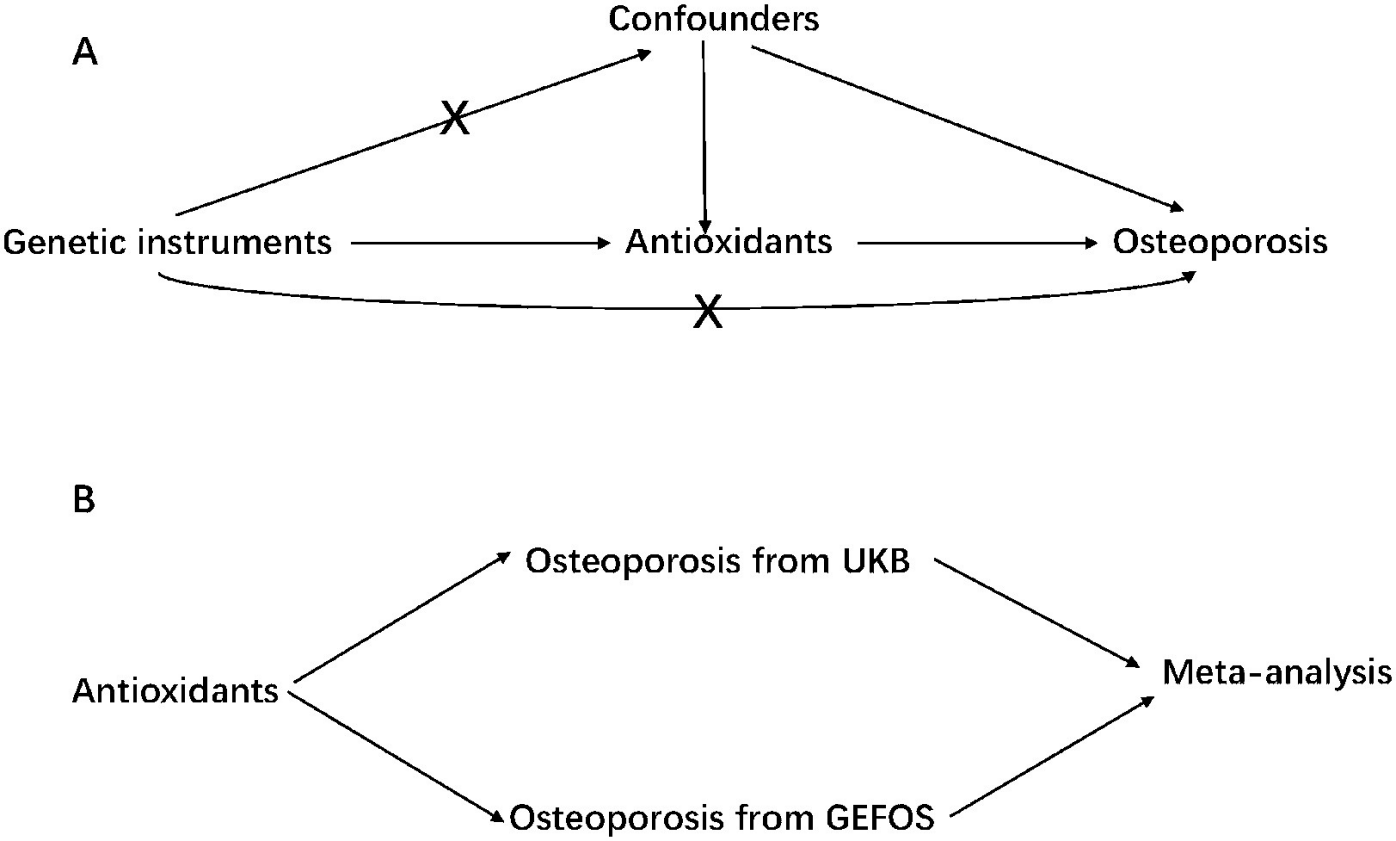
A. Basic principles of Mendelian randomization. B. Main design of the study. UKB, UK Biobank; GEFOS, Genetic Factors of Osteoporosis.

MR should be performed under three basic assumptions: (1) relevance criterion, whereby genetic variants are closely related to exposure; (2) independence criterion, where genetic variants should not be linked to any potential confounder that might affect the exposure-outcome relationship; (3) and exclusion-restriction criterion, where genetic variants should not be associated with the outcome when being conditioned on the exposure [18]. (Fig 1). Additional assumptions should also be satisfied, including linearity and no statistical interactions. We used the F statistic (F = beta^2^/se^2^) to evaluate the remaining genetic instrument power for each genetic instrument and calculated a general F statistic for all genetic instruments with the following formula:

$$\text{F}=\left(\left(\text{N}-\text{K}-1\right)/\text{K}\right)*\left({\text{R}}^{2}/\left(1-{\text{R}}^{2}\right)\right)$$

where N is the sample size of GWAS, K is the number of genetic instruments used, and R^2^ is the variance of exposure explained by the used instruments.

We tested the causal relationship between diet-derived antioxidants and osteoporosis using univariable MR analyses. We also estimated the causal effect of diet-derived antioxidants in each independent osteoporosis GWAS and then combined the MR results using meta-analysis in the forward MR as a comparison. However, osteoporosis is a dichotomous variable, which may lead to exclusion limit bias, because competing risk factors may be present in our study. To reduce exclusion limit bias, we estimated the causal effects of diet-derived antioxidants on BMD, which can be a surrogate. We did not use the GWAS meta-analysis results for some traits from UKB because of the sample overlap.

### Statistical analyses and data visualization

For MR analyses, we adopted the inverse variance-weighted (IVW) method as the main method to combine each IV effect size by R package “TwoSampleMR.” Moreover, we also used MR-Egger and weighted-median methods as supplements to IVW and used the Cochrane’s Q value to estimate heterogeneity. The MR-Egger intercept and MR-PRESSO methods were also used to detect horizontal pleiotropy [19]. If outliers were detected, they were removed and we would reassess the MR causal estimation. If heterogeneity still existed, we would adopt the median based estimation as the main effect size. The data visualization was performed by R package “ggforestplot.” All statistical analyses and data visualization were performed in R software 4.1.1 (https://www.r-project.org/).

## Results

### Result of osteoporosis in UKB consortium

**Fig 2:**
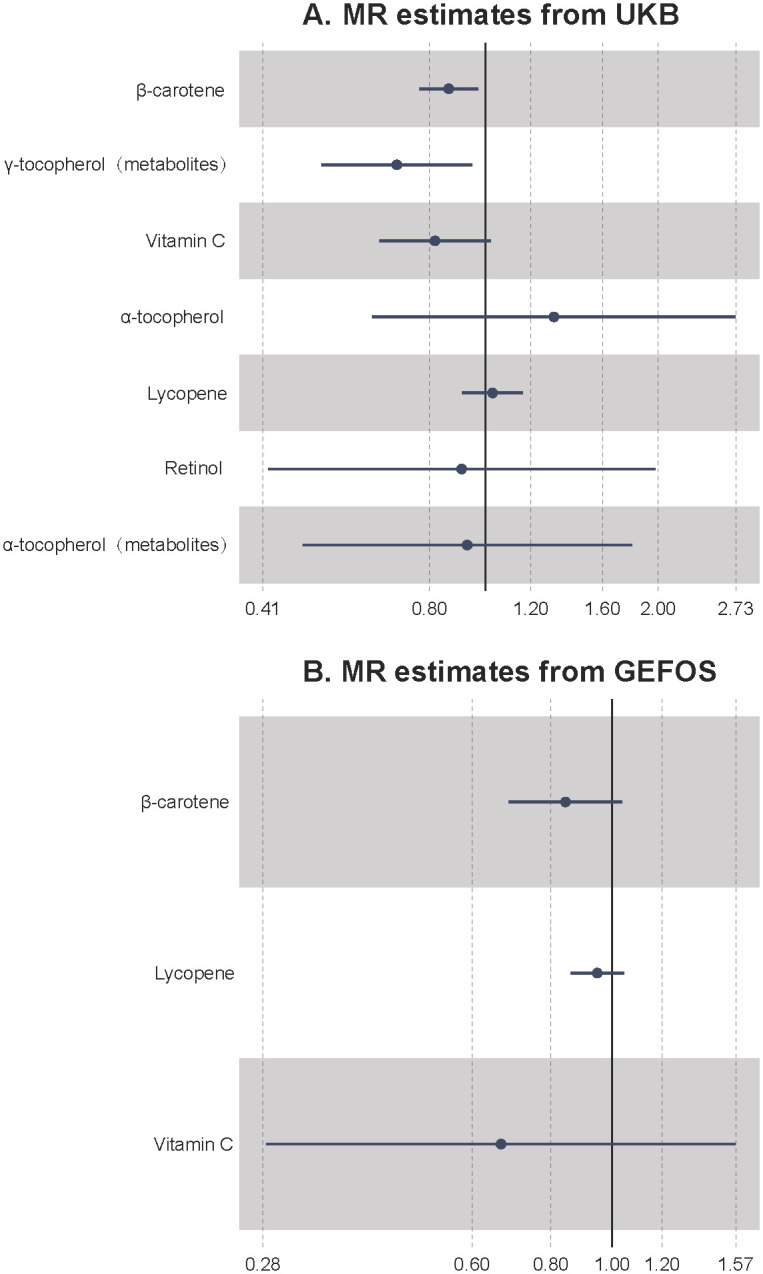
A. Mendelian randomization results from UKB. B. Mendelian randomization results from GEFOS. The x-axis represents the odds ratio. UKB, UK Biobank; GEFOS, Genetic Factors of Osteoporosis.

In the UKB data set, only the association of serum β-carotene with osteoporosis indicated statistical significance after FDR correction (OR = 0.863, p = 7.37 × 10^−6^, FDR = 5.16 x 10^−5^, power = 100%). The weighted-median method also suggested a significant inverse relationship between β-carotene and osteoporosis (OR = 0.846, p-value = 0.019, power = 100%). We had sufficient power to detect an 100% difference per mmol/L beta-carotene, which we considered reasonable and clinically relevant. Besides, the genetically predicted higher γ-tocopherol could suggestively decrease the risk of osteoporosis (p-value < 0.05). The odds of osteoporosis would decrease per 1-SD increase of γ-tocopherol (OR = 0.701, p = 0.021, power = 5%) (Fig 2A). The IVs showed 9.12% of serum β-carotene variance and the IVs of γ-tocopherol showed 4.14% of serum γ-tocopherol (Table 1). Since the number of IVs was <3, we did not test the horizontal pleiotropy for α- tocopherol, lycopene, and retinol. Besides, no horizontal pleiotropy and heterogeneity were found for other antioxidants. The results of the supplementary methods can be found in Tables 2 and 3. The association of β-carotene, α-tocopherol and osteoporosis is credible due to high powers, which are more than 80%. The association of γ-tocopherol, vitamin C and osteoporosis is underestimated due to low powers, which are less than 80%.

**Table 1:** Description of genetic instruments used in the Mendelian randomization study.

Exposure	NSNP	Unit	Sample	R^2^	F	PMID
β-carotene	5	μg/L in natural log-transformed scale	2,344	0.091	46.9339	23134893
γ-tocopherol metabolites	12	log10-transfomed metabolites concentration	5,822	0.041	20.88362	24816252
Vitamin C	10	per standard deviation	52,018	0.010	53.41919	33203707
α-tocopherol	3	mg/L in log-transformed scale	5,006	0.024	41.21858	21729881
Lycopene	2	μg/dL	441	0.130	32.70756	26861389
Retinol	2	μg/L in natural log-transformed scale	5,006	0.023	59.53746	21878437
α-tocopherol metabolites	8	log10-transfomed metabolites concentration	7,276	0.022	20.68115	24816252

NSNP, number of used single nucleotide polymorphisms in the analysis; R^2^, variance of exposure explained by the SNPs; F, F statistic calculated to evaluate the weak instrument bias; PMID, identifier of literature index in the PubMed database.

**Table 2:** Mendelian randomization results of weighted median.

	NSNP	Weighted median				POWER	P_heterogeneity_	P_pleiotropy_
		OR	95%LCI	95%UCI	P			
**UKB**								
α-tocopherol	3	1.180	0.416	3.344	0.755	100%		
α-tocopherol(metabolites)	8	1.212	0.512	2.866	0.662	100%	0.333	0.794
β-carotene	5	0.846	0.736	0.973	0.019	100%	0.877	0.576
γ-tocopherol(metabolites)	12	0.678	0.433	1.063	0.091	5%	0.418	0.551
Vitamin C	10	0.875	0.638	1.201	0.409	5%	0.290	0.421
**GEFOS**								
β-carotene	3	0.874	0.698	1.102	0.254	87.0%		
Lycopene	4	0.974	0.855	1.110	0.682	6.0%	0.413	0.472

UKB, UK Biobank; GEFOS, Genetic Factors of Osteoporosis; NSNP, number of used single nucleotide polymorphisms in the analysis; OR, odds ratio; 95% LCI, lower limit of 95% confidence interval; 95% UCI, upper limit of 95% confidence interval; P, p-value of odds ratio; P_heterogeneity_, p-value of heterogeneity test; P_pleiotropy_, p-value of horizontal pleiotropy test from MR-Egger intercept.

* In UKB results, the association of β-carotene, α-tocopherol and osteoporosis is credible due to high powers, which are more than 80%. The association of γ-tocopherol, vitamin C and osteoporosis is underestimated due to low powers, which are less than 80%.

* In GEFOS results, the association of β-carotene and osteoporosis is credible due to a high power, which is more than 80%. The association of lycopene and osteoporosis is underestimated due to a low power, which is less than 80%.

**Table 3:** Mendelian randomization results of MR-Egger.

	NSNP	MR-Egger				POWER	P_heterogeneity_	P_pleiotropy_
		OR	95%LCI	95%UCI	P			
**UKB**								
α-tocopherol	3	71.369	0.300	16980.786	0.369	100%		
α-tocopherol(metabolites)	8	0.692	0.073	6.550	0.759	100%	0.333	0.794
β-carotene	5	1.152	0.463	2.866	0.781	100%	0.877	0.576
γ-tocopherol(metabolites)	12	0.545	0.231	1.286	0.196	5%	0.418	0.551
Vitamin C	10	1.101	0.529	2.292	0.803	5%	0.290	0.421
**GEFOS**								
β-carotene	3	2.279	0.123	42.211	0.678	87.0%		
Lycopene	4	0.872	0.709	1.074	0.327	6.0%	0.413	0.472

UKB, UK Biobank; GEFOS, Genetic Factors of Osteoporosis; NSNP, number of used single nucleotide polymorphisms in the analysis; OR, odds ratio; 95% LCI, lower limit of 95% confidence interval; 95% UCI, upper limit of 95% confidence interval; P, p-value of odds ratio; P_heterogeneity_, p-value of heterogeneity test; P_pleiotropy_, p-value of horizontal pleiotropy test from MR-Egger intercept.

* In UKB results, the association of β-carotene, α-tocopherol and osteoporosis is credible due to high powers, which are more than 80%. The association of γ-tocopherol, vitamin C and osteoporosis is underestimated due to low powers, which are less than 80%.

* In GEFOS results, the association of β-carotene and osteoporosis is credible due to a high power, which is more than 80%. The association of lycopene and osteoporosis is underestimated due to a low power, which is less than 80%.

### Result of osteoporosis in the GEFOS consortium

In the GEFOS data set, only four antioxidants were included in the analysis, namely β-carotene, lycopene, vitamin C, and α-tocopherol. However, none were significant in this dataset (Fig 2B). The direction of the association of β-carotene with osteoporosis (OR = 0.844, p = 0.106, power = 87%) was consistent with that of the UKB dataset. The number of IVs used in GEFOS was fewer than that in the UKB dataset and the explained variance was correspondingly less. Thus, we deemed that such an insignificant result might be attributed to low statistical power in GEFOS, as it had fewer IVs and cases. The results of the supplementary methods can be found in Tables 2 and 3. The association of β-carotene and osteoporosis is credible due to a high power, which is more than 80%. The association of lycopene and osteoporosis is underestimated due to a low power, which is less than 80%.

### Combined results for osteoporosis by meta-analysis

**Fig 3:**
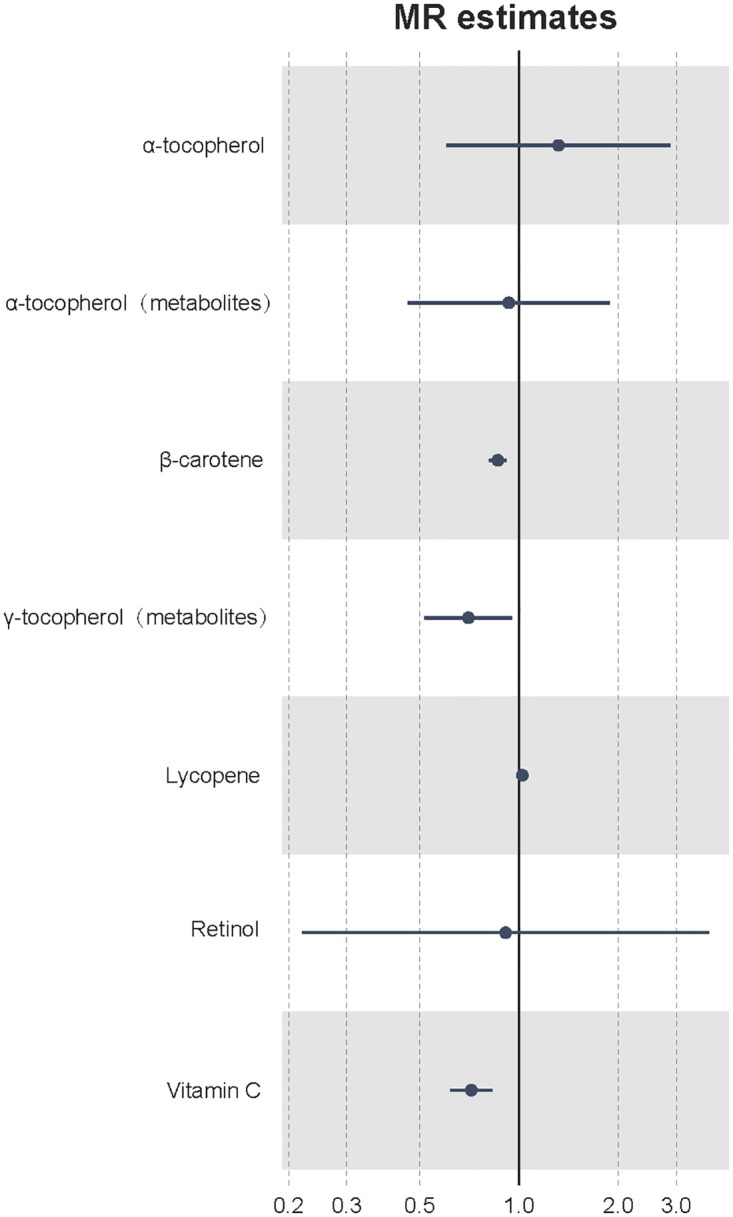
Meta-analysis results of Mendelian randomization estimates from UKB and GEFOS. The x-axis represents the odds ratio. UKB, UK Biobank; GEFOS, Genetic Factors of Osteoporosis.

Considering the relatively low statistical power of the GEFOS dataset, we combined the UKB and GEFOS dataset using a fixed-effects meta-analysis model. The meta-analysis of MR results from UKB and GEFOS further confirmed that previous antioxidants could decrease the risk of osteoporosis, including β-carotene (OR = 0.862, p = 2.21 × 10^−6^) and γ-tocopherol (OR = 0.701, p = 2.31 × 10^−2^) (Fig 3). No heterogeneity was found in the meta-analysis (Cochran’s Q p-value = 0.844). The combined results of β-carotene from the weighted-median method suggested a significant association (OR = 0.854, p-value = 0.011) without heterogeneity (Cochran’s Q p-value = 0.818).

### Result of total body BMD, lumbar spine BMD and femoral neck BMD in the GEFOS consortium

**Fig 4:**
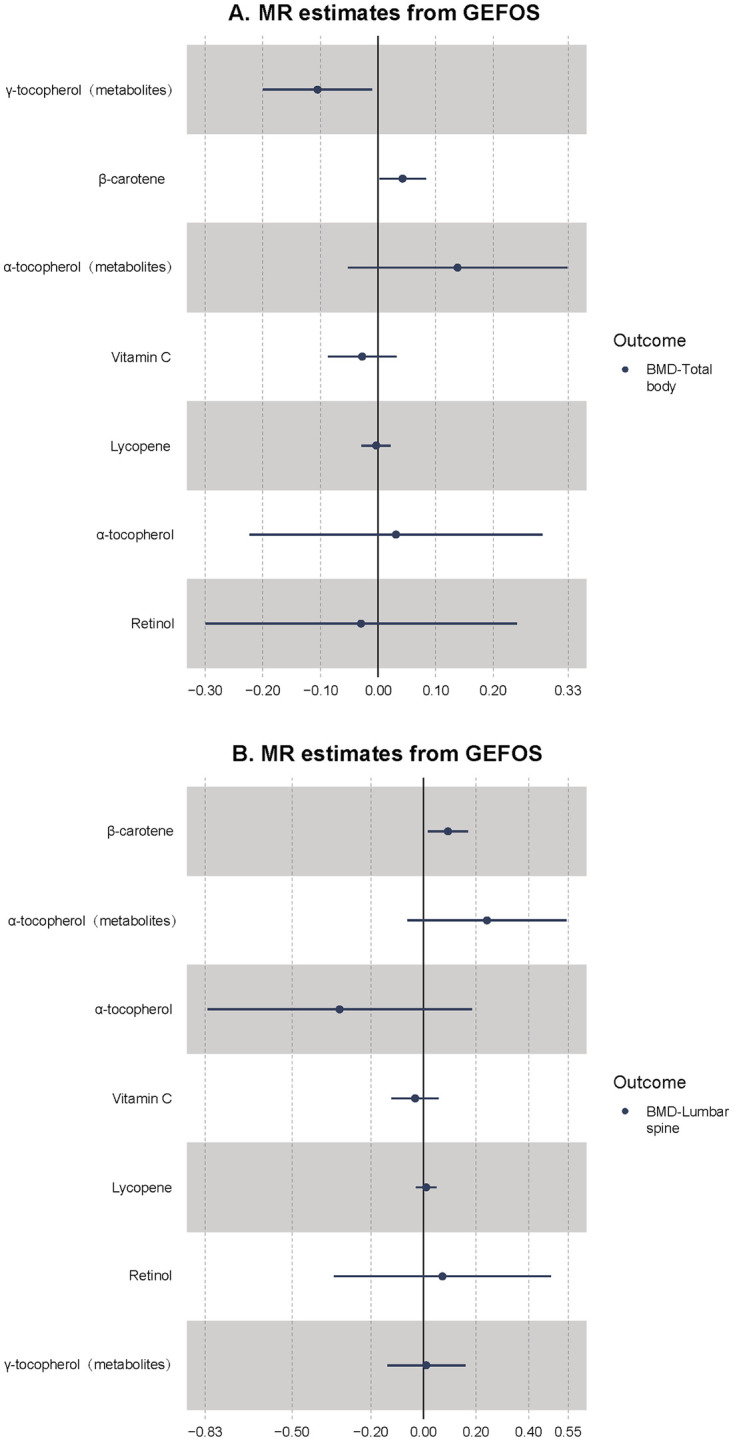
A. Mendelian randomization results of total body BMD. B. Mendelian randomization results of lumbar spine BMD. The x-axis represents the beta value. UKB, UK Biobank; GEFOS, Genetic Factors of Osteoporosis.

**Fig 5:**
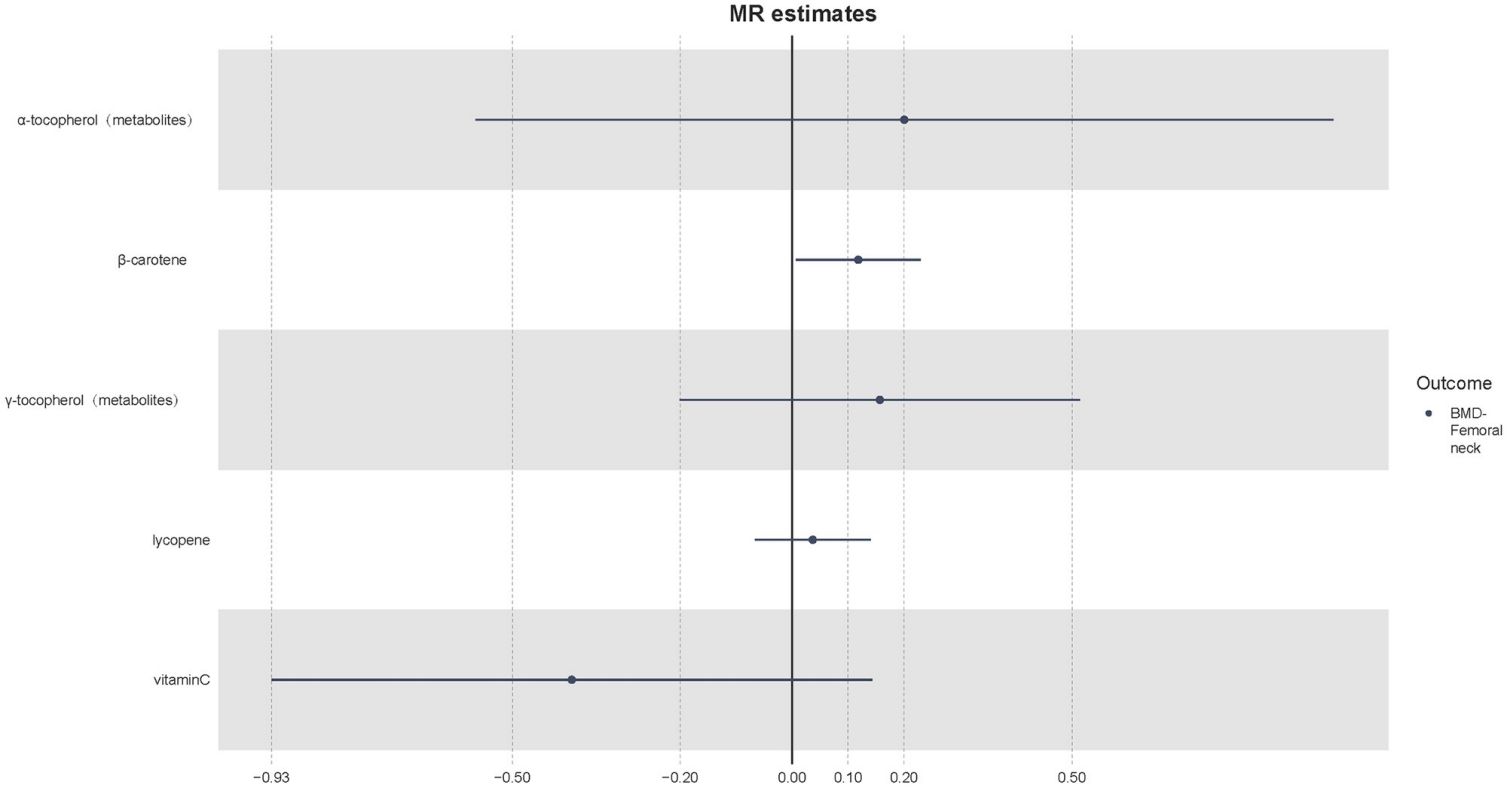
Mendelian randomization results of femoral neck BMD. The x-axis represents the beta value. UKB, UK Biobank; GEFOS, Genetic Factors of Osteoporosis.

Considering BMD is a continuous variable, we adopted “beta” to evaluate the effect size. In the GEFOS dataset, we found that the genetically elevated circulating β-carotene level could increase total body BMD (beta = 0.043, p-value = 8.26 x 10^−5^, power = 100%) (Fig 4A). No horizontal pleiotropy and heterogeneity were found for β-carotene (Cochran’s Q p-value = 0.894; MR-Egger intercept p-value = 0.537). The genetically elevated circulating β-carotene level could also increase lumbar spine BMD (beta = 0.226, p-value = 0.001, power = 100%) (Fig 4B) and femoral neck BMD (beta = 0.118, p-value = 0.016, power = 100%) (Fig 5). In addition, we found significant heterogeneity (Cochran’s Q p-value = 0.001) and outliers for the association of vitamin C with total body BMD; however, the result was not significant after removal of outliers (beta = -0.017, p-value = 0.586, power = 8%). Besides, the GEFOS results did not suggest that genetic liability to γ-tocopherol could promote total body BMD (beta = -0.105, p-value = 0.087, power = 13%). The associations of β-carotene and total body BMD, lumbar spine BMD and femoral neck BMD are credible due to high powers, which are more than 80%.

## Discussion

Our MR study unveils the inverse association of genetically predicted serum levels of β-carotene with the risk of osteoporosis and indicates that the dietary supplementation thereof might help to elevate BMD and reduce the risk of osteoporosis. We found no robust causal associations of other antioxidants with osteoporosis risk.

Oxidative stress has been established as a pivotal risk factor in the pathogenesis of osteoporosis, and the reduction of reactive oxygen species (ROS) has been proposed to address the development of this disease [3, 20]. Thus, antioxidants, especially dietary ones, should have a role in this aspect. Prior studies have noted the importance of β-carotene in ameliorating osteoporosis. Regu et al. found that postmenopausal women in the highest quintile of daily β-carotene intake had a lower risk of osteopenia at the lumbar spine in a cross-sectional study of 8,022 Korean adults (3,763 males and 4,259 females) [21]. The result of that study indicate that β-carotene should have its role in protecting people against osteoporosis, which lends support to our MR findings. Kan et al. performed a cross-section study in 4,820 US individuals with an average age ≥ 50 and found that people whose average age was 61.9 in the highest quintile of daily β-carotene intake had a lower risk of osteoporosis [22]. Additionally, a 1:1 matched case-control study recruited 726 elderly Chinese with hip fractures and 726 control subjects, and showed that a higher dietary intake of β-carotene is associated with a lower risk of hip fracture in elderly Chinese people [23]. Mechanistically, β-carotene is the most important source of non-preformed vitamin A and contains a series of conjugated double bonds, which makes it susceptible to oxidative and enzymatic cleavage. Therefore, β-carotene has direct antioxidant effects, including quenching singlet oxygen and lipid peroxides [24–28]. Additionally, β-carotene can be cleaved to form vitamin A, which acts through its metabolite, all-trans-retinoic acid (ATRA), a highly potent transcriptional regulator which can affect the transcription of genes that are critical to mediate the indirect antioxidant effects of vitamin A. Therefore, vitamin A can activate the NRF2/KEAP1 or NF-κB signaling pathways to inhibit oxidative stress as an indirect antioxidant [29, 30], and β-carotene and its metabolite can increase BMD and reduce the risk of osteoporosis via both direct and indirect antioxidant effects. Therefore, we can increase β-carotene intake as preventative and therapeutic intervention to prevent osteoporosis and improve BMD. We could encourage a diet rich in antioxidants especially β-carotene and an antioxidant-preserving lifestyle along with other traditional therapeutic regimens to help patients with osteoporosis. Besides, Larger clinical trials are needed to make clinical recommendations credible.

Our results also suggest that a genetically elevated serum γ-tocopherol level is marginally associated with a reduced risk of osteoporosis in the UKB dataset (p-value < 0.05). γ-Tocopherol is the second most predominant isoform of vitamin E in dietary sources, consisting of 20–25% of consumed vitamin E, and it can scavenge both reactive nitrogen species (RNS) and ROS, which is different from α-tocopherol [31]. Thus, γ-tocopherol, instead of α-tocopherol, can help reduce the risk of osteoporosis. A clinical trial suggested that γ-tocopherol supplementation decreases systemic oxidative stress, but cross-sectional studies differed. Yang et al. did not find significant results between γ-tocopherol and BMD in over 5,000 older women [32]; however, Ilesanmi-Oyelere et al. discovered that a higher intake of vitamin E is associated with a decreased level of BMD [33]. Additionally, an RCT with 52 participants implied that vitamin E supplementation in postmenopausal women with osteopenia may have a preventive effect on bone loss [34]. RCTs usually elicit higher graded evidence of causality, and our MR findings appear to support that study with an enlarged sample size. Many studies have suggested that vitamin E inhibits the propagation of the chain reaction of lipid peroxidation to scavenge ROS. Therefore, γ-tocopherol can only inhibit oxidative stress as a direct antioxidant, which should be helpful to explain why the effect of γ-tocopherol is weaker in reducing the risk of osteoporosis than that of β-carotene even though they are both antioxidants. There is limited evidence to date on the effects of γ-tocopherol on osteoporosis risk factors. Therefore, Higher-quality trials are required.

In our study, the increase of other antioxidants including lycopene, retinol, vitamin C, and α-tocopherol could not decrease the risk of osteoporosis. Lycopene is a type of carotenoid, which is different from β-carotene, and is a non-provitamin A carotenoid. Therefore, lycopene cannot be cleaved to form vitamin A to inhibit oxidative stress as an indirect antioxidant, and can only inhibit oxidative stress directly, resulting in the weaker antioxidant capacity than that of β-carotene. A multicenter RCT with 198 postmenopausal Indian women found that lycopene supplementation might protect against osteoporosis [35]. However, a cross-sectional study with 4,820 individuals from the United States found that there was no association of lycopene intake with osteoporosis [22]. There may be an undetected weak instrument and insufficient statistical effectiveness in our study, resulting in negative results. Retinol is vitamin A, which has the effect of anti-oxidation as well as toxicity. Intake from preformed sources of vitamin A usually exceeds the recommended dietary allowances (RDA) for adults [36]. Four prospective, observational studies have suggested that chronic high intakes of preformed vitamin A are associated with bone loss, leading to osteoporosis and hip fractures [37–40]. Arctic people traditionally have high intakes of preformed vitamin A, and reports regarding their skeletal remains and toxicity confirm bone involvement [41, 42]. Several studies imply that α-tocopherol has conflicting effects on bone health; further, α-tocopherol is the most abundant vitamin E isomer present in food and the most widely distributed in our body [43, 44]. Chan et al. found that the intake of α-tocopherol is positively correlated with total BMD at the spine in two groups of young adult women in China (n = 441; age range: 25–30 years) [45]. However, MacDonald et al. discovered that the intake of α-tocopherol from diet alone was negatively associated with BMD at the lumbar spine and femoral neck in a longitudinal study with a group of early menopausal women (n = 891 women; age range at baseline = 45–55 years; age range at follow-up = 50–59 years) [46]. The high intake of α-tocopherol might be detrimental to bone in three ways, including interfering with vitamin K function upon bone, blocking the entry of other vitamin E isomers beneficial to bone, and playing a role as a pro-oxidant [47]. Thus, the detrimental and beneficial effects of α-tocopherol should be canceled out in the MR analysis. Evidence from our study did not support a beneficial role of circulating dietary-derived lycopene, retinol or α-tocopherol on osteoporosis risk in the general population. This signifies the absence of a substantial role of these dietary-derived antioxidants on osteoporosis risk.

Vitamin C, also known as L-ascorbic acid, is a water soluble vitamin which is often considered to be beneficial to human bone health [48]. Vitamin C is able to induce osteoblast and osteoclast formation *in vitro*, but can also be cytotoxic to both at high doses [49]. New et al. found that there was a U-shaped relationship between vitamin C intake and BMD in the Osteoporosis Screening Program of 994 healthy premenopausal women aged 45–49 years in Aberdeen [50]. Simon et al. found that serum vitamin C level had a significant association with hip BMD in men (n = 6,137, mean age = 44 ± 16 years) in a nonlinear fashion in the Third National Health and Nutrition Examination Survey (NAHANES III) [51]. Hip BMD increased with the increase of serum vitamin C first, then decreased with a continued rise of serum vitamin C levels [51]. There was also a similar U-shaped relationship between vitamin C intake and self-reported fracture probability among men. Fracture probability decreased with the increase of serum vitamin C first, then increased with a continued rise of serum vitamin C levels [51]. Currently, our study could not provide evidence to suggest that Vitamin C reduces the risk of osteoporosis. Therefore, Higher-quality trials are required to determine whether Vitamin C has a substantial effects on the risk of osteoporosis.

The strengths of our study can be claimed as follows. First, our study is an MR design, which is suitable for causal inference and can avoid reverse causation with fewer economic costs. Second, the sample size in our study is large and the results are replicated in independent datasets, which can compensate for the limited sample size of RCTs. Third, several complementary methods and sensitivity analyses have been applied, guaranteeing the robustness of our results. Finally, the participants of all GWAS studies were primarily from European ancestry and all studies have genomic control, indicating that population stratification and genomic inflation are unlikely to bias our results.

However, there are still several limitations in this MR study, which should be noted. The greatest concern in the MR setting is pleiotropy, which can be classified into vertical and horizontal pleiotropy. Vertical pleiotropy means that the SNP influences one trait (exposure), which in turn influences another (outcome), and usually represents the MR estimates. Horizontal pleiotropy means that the SNP influences two traits in dependently. Vertical pleiotropy can be tested by MR analysis, whereas the horizontal pleiotropy should be avoided in MR. Although we use two main methods to detect horizontal pleiotropy, which are MR-Egger intercept and MR-PRESSO [19], it cannot be completely eliminated in an MR study. We hope the methods can minimize the bias caused by horizontal pleiotropy. In addition, osteoporosis is a dichotomous variable, which may lead to exclusion limit bias, because competing risk factors may be present in our study. Therefore, we used BMD as a surrogate to reduce bias. Due to the unavailability of individual-level data, we cannot evaluate the nonlinear relationship between serum antioxidants and BMD. Lastly, care should be taken when expanding our conclusions to other populations, because the participants of the included GWAS studies were primarily Europeans.

## Conclusion

This MR study demonstrated that high β-carotene intake may be beneficial to prevent osteoporosis and elevate BMD of total body and lumbar spine.

## Abbreviations

ATRA: All-trans-retinoic acid
BMD: Bone mineral density
BMI: Body mass index
GEFOS: Genetic Factors of Osteoporosis
MR: Mendelian randomization
NHANES III: Third National Health and Nutrition Examination Survey
RCT: Randomized controlled trial
RDA: Recommended dietary allowances
RNS: Reactive nitrogen species
ROS: Reactive oxygen species
UKB: UK Biobank
